# Single-Molecule FISH Reveals Non-selective Packaging of Rift Valley Fever Virus Genome Segments

**DOI:** 10.1371/journal.ppat.1005800

**Published:** 2016-08-22

**Authors:** Paul J. Wichgers Schreur, Jeroen Kortekaas

**Affiliations:** Department of Virology, Central Veterinary Institute, part of Wageningen University and Research Centre, Lelystad, The Netherlands; Icahn School of Medicine at Mount Sinai, UNITED STATES

## Abstract

The bunyavirus genome comprises a small (S), medium (M), and large (L) RNA segment of negative polarity. Although genome segmentation confers evolutionary advantages by enabling genome reassortment events with related viruses, genome segmentation also complicates genome replication and packaging. Accumulating evidence suggests that genomes of viruses with eight or more genome segments are incorporated into virions by highly selective processes. Remarkably, little is known about the genome packaging process of the tri-segmented bunyaviruses. Here, we evaluated, by single-molecule RNA fluorescence *in situ* hybridization (FISH), the intracellular spatio-temporal distribution and replication kinetics of the Rift Valley fever virus (RVFV) genome and determined the segment composition of mature virions. The results reveal that the RVFV genome segments start to replicate near the site of infection before spreading and replicating throughout the cytoplasm followed by translocation to the virion assembly site at the Golgi network. Despite the average intracellular S, M and L genome segments approached a 1:1:1 ratio, major differences in genome segment ratios were observed among cells. We also observed a significant amount of cells lacking evidence of M-segment replication. Analysis of two-segmented replicons and four-segmented viruses subsequently confirmed the previous notion that Golgi recruitment is mediated by the Gn glycoprotein. The absence of colocalization of the different segments in the cytoplasm and the successful rescue of a tri-segmented variant with a codon shuffled M-segment suggested that inter-segment interactions are unlikely to drive the copackaging of the different segments into a single virion. The latter was confirmed by direct visualization of RNPs inside mature virions which showed that the majority of virions lack one or more genome segments. Altogether, this study suggests that RVFV genome packaging is a non-selective process.

## Author Summary

The bunyavirus family is one of the largest virus families on Earth, of which several members cause severe disease in humans, animals or plants. Little is known about the mechanisms that facilitate the production of infectious bunyavirus virions, which should contain at least one copy of the small (S), medium (M) and large (L) genome segment. In this study, we investigated the genome packaging process of the Rift Valley fever virus (RVFV) by visualizing individual genome segments inside infected cells and virions. Experiments performed with wild-type virus, two- and four-segmented variants, and a variant with a codon-shuffled M segment showed that the production of infectious virions is a non-selective process and is unlikely to involve the formation of a supramolecular viral RNA complex. These observations have broad implications for understanding the bunyavirus replication cycle and may facilitate the development of new vaccines and the identification of novel antiviral targets.

## Introduction

Rift Valley fever virus (RVFV) is a zoonotic bunyavirus of the genus *Phlebovirus* that causes recurrent outbreaks on the African continent, the Arabian Peninsula and several islands off the coast of Southern Africa. The virus predominantly affects ruminants, of which sheep are the most severely affected. Epizootics are characterized by massive abortions of pregnant ewes and high mortalities among newborns. Infected humans generally display mild flu-like symptoms, however in a minority of cases severe complications such as retinitis, hemorrhagic fever, and delayed-onset encephalitis may develop [1]. In humans, the overall case fatality ratio is estimated to range from 0.5 to 2%. Mosquito vectors of the *Aedes* and *Culex* genera are associated with RVFV transmission in endemic areas and are also present in other regions of the world with high ruminant density.

Like all bunyaviruses, RVFV contains a tri-segmented single-stranded RNA genome of negative polarity [2]. The large (L), medium (M) and small (S) genome segments are encapsidated by the nucleocapsid (N) protein, which is translated from a subgenomic mRNA transcribed from the genomic-sense S RNA. Encapsidated genome segments are referred to as ribonucleoproteins (RNPs). The antigenomic-sense S-segment additionally encodes the non-structural protein NSs. NSs is the main virulence factor of the virus and is known to antagonize host innate immune responses [3–5]. The M-segment encodes the two major structural glycoproteins Gn and Gc [6] which are involved in host cell entry and fusion, respectively. The M-segment also encodes two accessory proteins, known as NSm and 78-kDa protein. NSm was shown to have anti-apoptotic function [7,8] and the 78-kDa protein was shown to be incorporated predominantly into virions matured in insect cells [9]. The L-segment encodes the RNA-dependent RNA polymerase, which is responsible for transcription of genes and replication of the viral genome [10]. Remarkably, and in contrast to many other RNA viruses, bunyavirus mRNA synthesis is coupled to translation to prevent premature transcription termination [11]. The termini of all bunyavirus genome segments are inverted complementary and facilitate the formation of a panhandle structure, which comprises signals for transcription, replication and encapsidation [12–19].

Bunyavirus particles assemble in so called ‘virus factories’, located at the Golgi network [20–23]. In these factories viral budding is believed to be initiated by interactions of the RNPs with the cytoplasmic tail of the Gn protein [19,22,24,25]. How infectious particles, containing at least one S, one M and one L RNP, assemble is not yet fully understood. Interestingly, in 2011 Terasaki and co-workers provided some clues for a selective genome packaging process using a virus-like particle (VLP) system. They suggested that copackaging of S, M and L genome segments into individual RVFV virions is mediated by direct or indirect inter-segment interactions, with a central role for the M-segment [17]. Other findings however suggest that inter-segment interactions do not play a major role in RVFV genome packaging. A fully viable two-segmented RVFV variant lacking the M-segment was described [26] and RVFV replicon particles that comprise only S and L genome segments can be produced very efficiently [27,28]. More recent results further emphasize the flexibility of the RVFV genome. A RVFV variant with a ‘swapped’ S segment, encoding N from the NSs locus and *vice versa*, is viable [29]. Moreover, four-segmented RVFV variants were recently created, which may contain two or even three M-type segments [30].

Here, we investigated the RVFV genome packaging process using state-of-the-art fluorescence *in situ* hybridization (FISH). Experiments with infected cells and mature virions revealed that copackaging of all three genome segments into individual particles is unlikely to involve the formation of a supramolecular complex. Instead, our results reveal that RVFV genome packaging is a non-selective process.

## Results

### Visualization of RVFV genome replication and recruitment in time

To investigate replication and recruitment of the RVFV RNA genome segments inside an infected cell, a single-molecule multicolor RNA FISH assay was developed. Probes were designed to be complementary to the S, M and L viral RNAs. After confirming specificity and optimizing sensitivity (S1 Fig), the assay was used to evaluate the genome segment distribution in RVFV-infected Vero cells at 2, 4, 6, 8 and 10 hours post infection (hpi).

**Fig 1:**
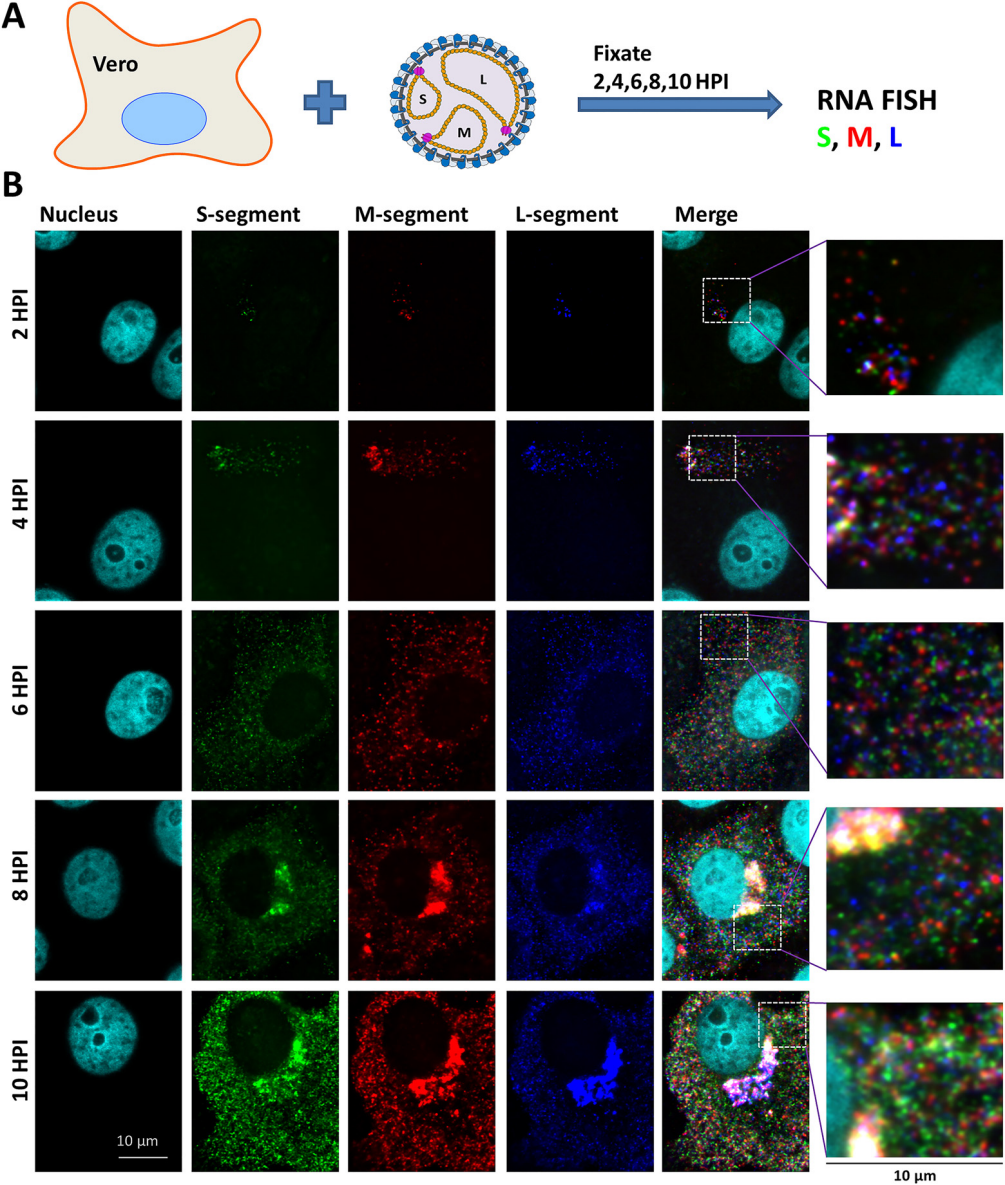
Single molecule vRNA FISH of RVFV infected cells in time. (**A**) Schematic presentation of the experimental design. (**B**) Vero cells were infected at MOI 0.1 with RVFV and cells were fixed at 2,4,6,8 and 10 hpi. Cells were subsequently probed against the S segment (N gene) using fluorescein labelled probes (green), against the M segment (Gn gene) using quasar 670 labelled probes (red) and against the L-segment (polymerase gene) using quasar 570 labelled probes (blue). Cell nuclei were visualized with dapi (cyan). Images of individual cells were taken using a wide-field microscope. Magnified images of the squared regions are shown at the right of each panel. The merged images show the spatial relationship between all the different channels.

**Fig 2:**
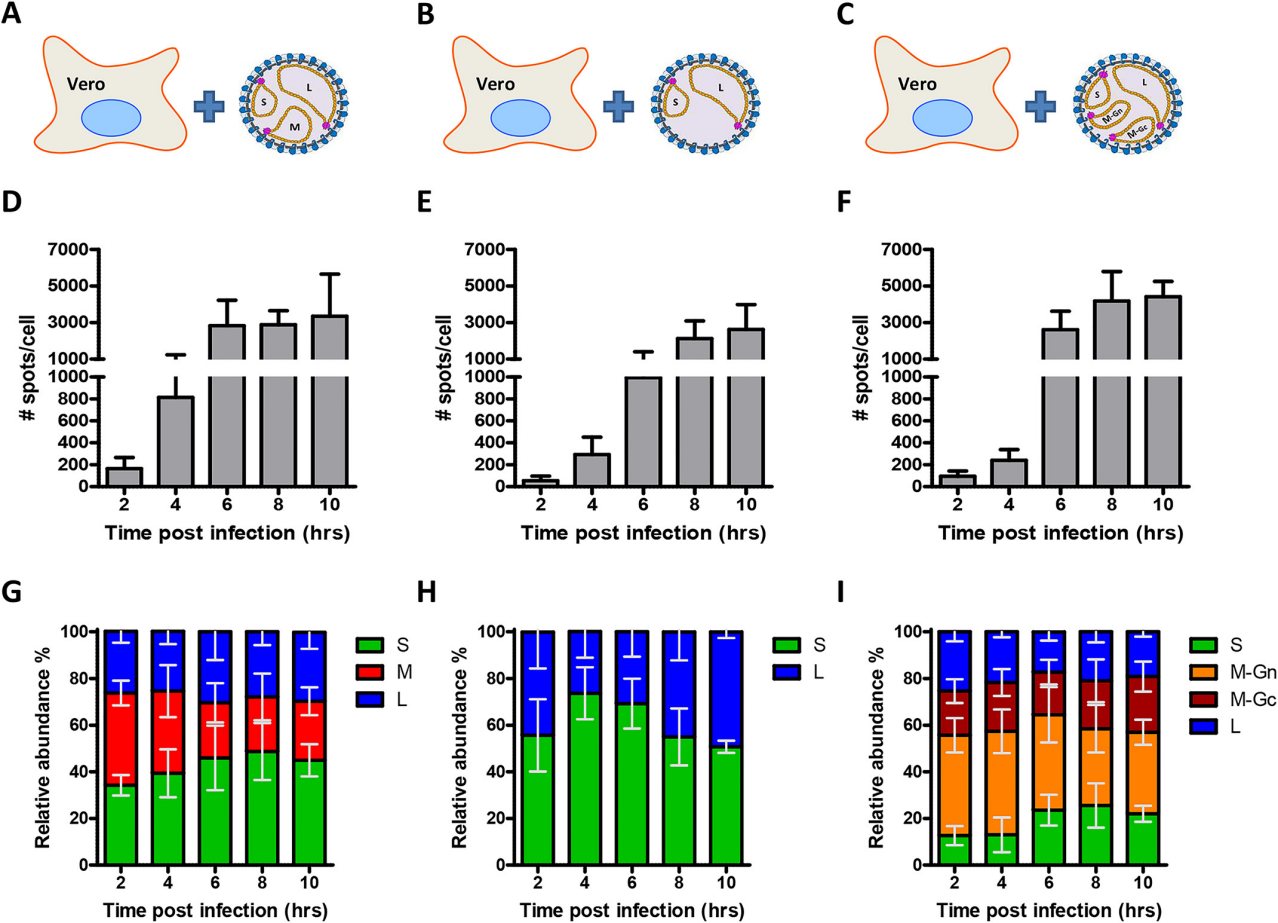
Quantification of cytoplasmic vRNAs of RVFV, NSR and RVFV-4s infected cells by single molecule FISH. Vero cells were infected at MOI 0.1 with RVFV (A), NSR (B) or RVFV-4s (C) and cells were fixed at 2,4,6,8 and 10 hpi. RVFV infected cells were subsequently probed against the S segment (N gene) using fluorescein labelled probes, against the M segment (Gn gene) using quasar 670 labelled probes and against the L-segment (polymerase gene) using quasar 570 labelled probes. NSR infected cells were probed against the S segment (N gene, green) using quasar 670 labelled probes and against the L-segment (polymerase gene) using quasar 570 labelled probes. RVFV-4s infected cells were either probed against the S segment (N gene) using fluorescein labelled probes, against the M-Gn segment (Gn gene) using quasar 670 labelled probes and against the L-segment (polymerase gene) using quasar 570 labelled probes or against the S segment (N gene) using fluorescein labelled probes, against the M-Gc segment (Gc gene) using quasar 670 labelled probes and against the L-segment (polymerase gene) using quasar 570 labelled probes. After image acquisition using a widefield microscope, spots were counted as described in the M&M section. The total number of spots (sum spots of all channels) and the relative abundance of each genome segment (in % of total) in the cytoplasm is calculated. The total number of spots (D,E,F) and relative abundance (G,H,I) of each genome segment at the cytoplasm at the indicated time points for RVFV, NSR and RVFV-4s infected cells are indicated. Spot counting data was obtained from >8 cells per experimental variable and means and SDs are presented.

At 2 and 4 hpi a single patch of up to 600 genome segments was detected in the cytoplasm of most infected cells (Figs 1 and 2D). The location of the patch varied among cells and at higher multiplicity of infection (MOI) cells with more than one patch of genome segments were observed as well. Most likely, the genome segments of an infecting virion start to replicate near the site of infection immediately after fusion of the viral membrane with the endosomal membrane.

At about 4–6 hpi the total level of genome segments had increased considerably and a more random cytoplasmic distribution of genome segments was observed. The average doubling time of a genome segment was estimated to be about 40 min. At 6–8 hpi recruitment of genome segments to the virion assembly site at the Golgi [20–23,31,32] became evident in most of the infected cells. The total level of cytoplasmic genome segments reached a plateau around 6 hpi, which is probably the result of ongoing replication and continuous Golgi recruitment and budding of particles containing mature RNPs. An average cytoplasmic inter-segment ratio approaching 1:1:1 between the S, M and L segments was observed during the first 4 hrs of infection, whereas later on, due to more efficient Golgi recruitment of the M-segment, the cytoplasmic ratios slightly changed (Fig 2G).

**Fig 3:**
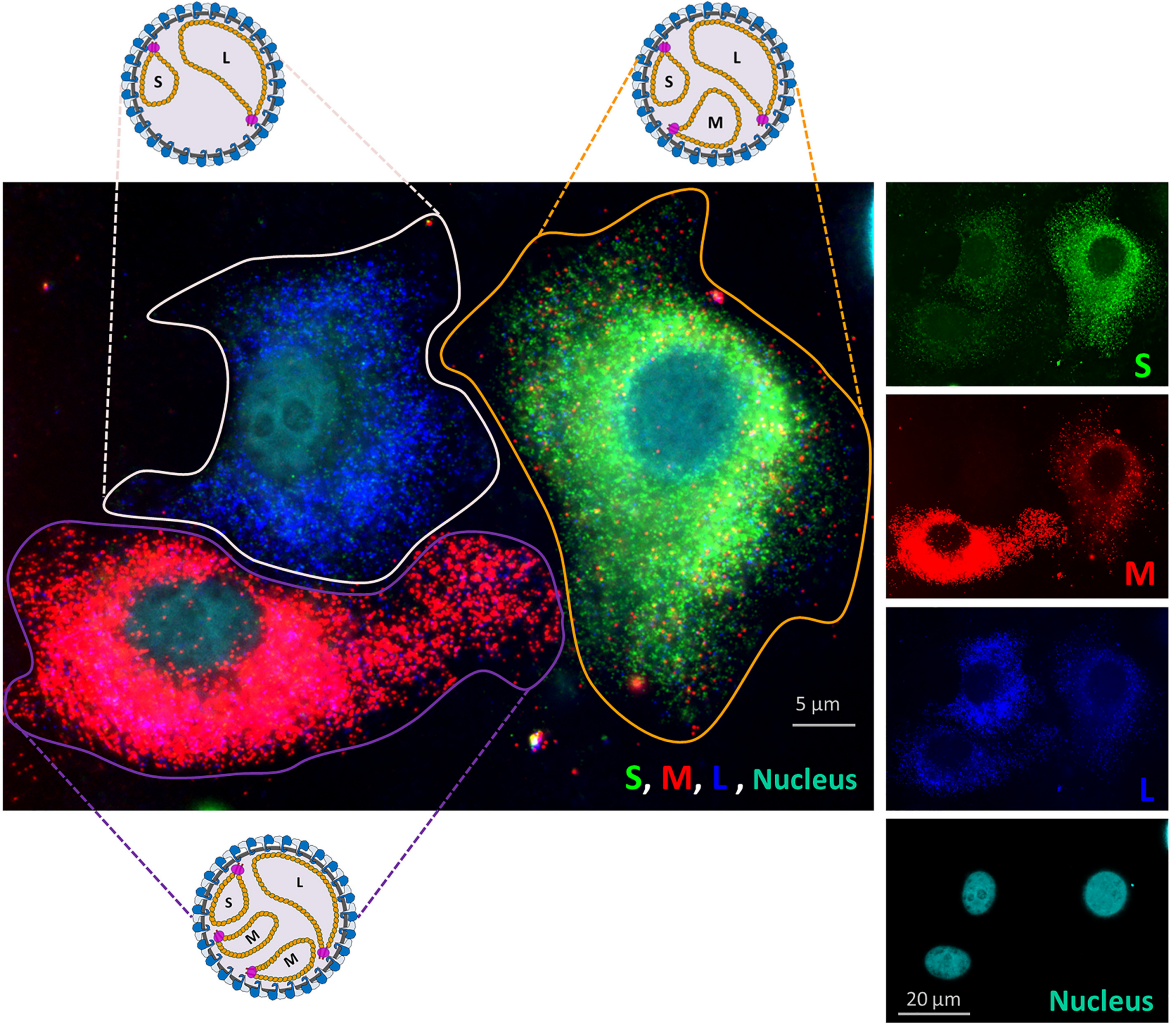
Single molecule vRNA FISH of multiple RVFV infected cells. RVFV infected Vero cells (MOI 0.1) were fixed at 7 hpi. Cells were subsequently probed against the S segment (N gene) using fluorescein labelled probes (green), against the M segment (Gn and Gc gene) using quasar 670 labelled probes (red) and against the L-segment (polymerase gene) using quasar 570 labelled probes (blue). Cell nuclei were visualized with dapi (cyan). The picture shows that the molar ratios of different vRNAs vary among cells. Most likely the presented cells were infected with either a particle containing a single copy of each genome segment, a particle lacking the M-segment, and a particle with an additional M-segment, respectively. In S2 Fig, additional images are presented.

Remarkably, in about 30–40% of infected cells the segment ratios were strikingly different. Cells with about twice as many S, M or L segments as well as cells lacking any evidence of M-segment replication (up to 25%) were observed frequently (Fig 3 and S2 Fig). It is important to note that cells infected with particles lacking an S and/or L segment will not reveal genome replication and are not detected by FISH. Altogether these results suggest that during particle assembly no quality control mechanisms are present that ensure packaging of each type of genome segment.

### RVFV RNPs do not form a supramolecular complex in the cytoplasm

**Fig 4:**
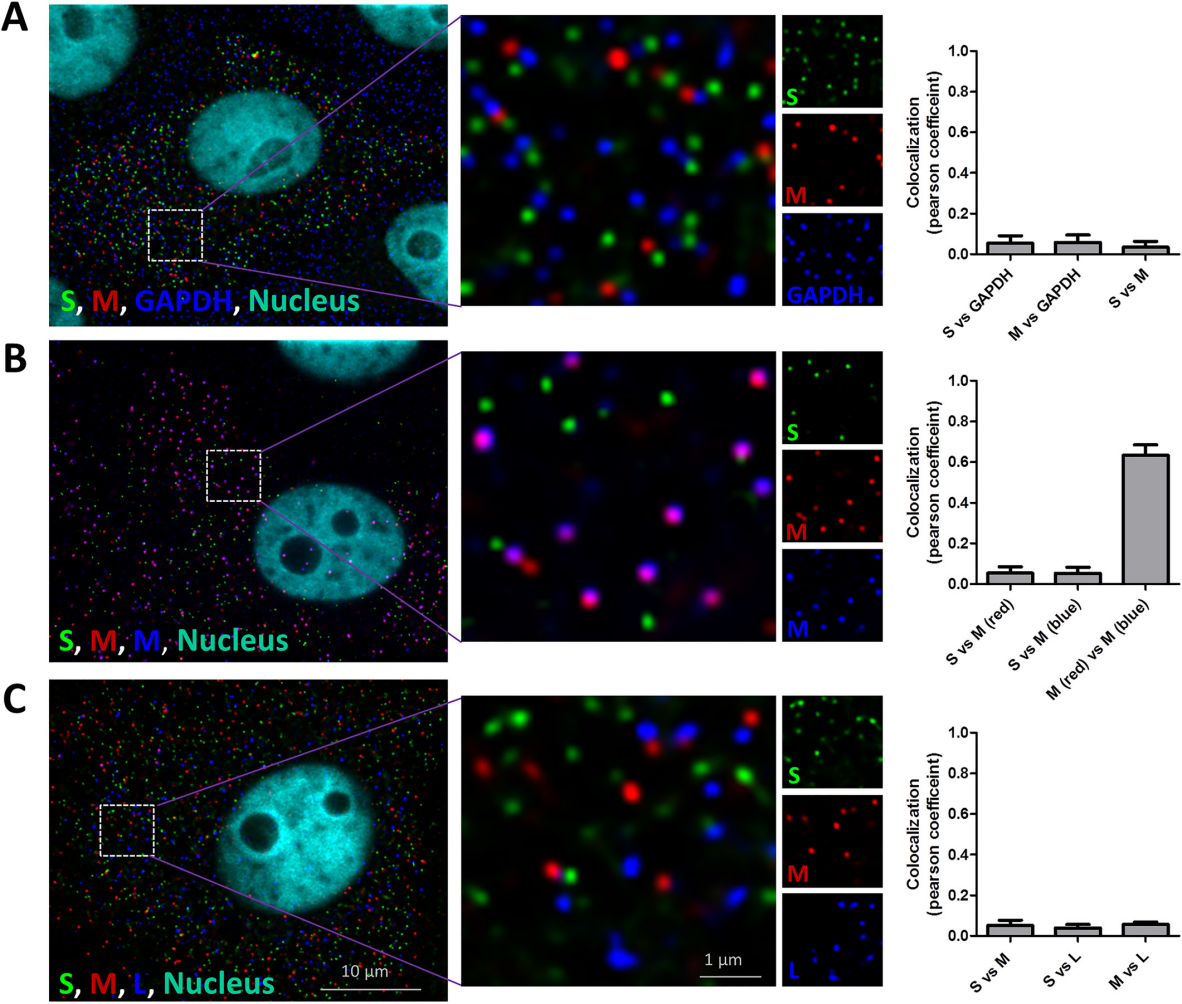
Colocalization coefficient of vRNAs in RVFV infected cells. Vero cells were infected at MOI 0.1 with RVFV and cells were fixed at 5 hpi. Cells were subsequently probed against (A) the S segment (N gene, green) using fluorescein labelled probes, the M segment (Gc gene, red) using quasar 670 labelled probes and the GAPDH mRNA using quasar 570 labelled (blue) probes or against (B) the S segment (N gene, green) using fluorescein labelled probes, the M segment (Gc gene, red) using quasar 670 labelled probes and the M-segment using quasar 570 labelled (Gn gene, blue) probes or against the (C) S segment (N gene, green) using fluorescein labelled probes, against the M segment (Gn gene, red) using quasar 670 labelled probes and against the L-segment (polymerase gene, blue) using quasar 570 labelled probes. Cell nuclei were visualized with dapi (cyan). Images were taken using a wide-field microscope. The level of colocalization is determined by calculation of the Pearson’s colocalization coefficient. Bars represent means and SDs of 4 independent measurements.

To evaluate whether S, M and L genome segments form a supramolecular complex and comigrate to the Golgi prior virion assembly we evaluated the extent of S, M and L colocalization at 5 hpi. The 5 hpi time point was selected because at this stage of infection the genome segment density was relatively high and the resolution of spots, corresponding to single genome segments, was still sufficient to discriminate between colocalized spots and non-colocalized spots. Moreover, Golgi recruitment has not yet started at this time point. As a positive colocalization control, cells were probed with two differentially labelled probe sets recognizing either the Gn or Gc gene, which are both encoded by the M genome segment. As a negative control, cells were probed with a GAPDH mRNA probe set and a Gc probe set. The Pearson colocalization coefficient of the probe sets recognising either the Gn or Gc-coding region was on average 0.65 and the colocalization coefficient of the GAPDH and Gc probe sets was below 0.1 (Fig 4A and 4B). These values, which are similar to what others have reported in the influenza field [33], confirm that our FISH assay is well suited for studying genome segment colocalization.

The Pearson colocalization coefficients of the different RVFV genome segments were all below 0.1 (Fig 4C). This indicates that RVFV genome segments, in contrast to the genome segments of the influenza virus [33,34], do not form a supramolecular complex consisting of more than one genome segment in the cytoplasm.

### RVFV glycoproteins play an important role in genome segment recruitment

The important role of the RVFV glycoproteins, specifically the cytoplasmic tail of the Gn protein, in RNP incorporation into virions is well recognized [19,22]. The involvement of the glycoproteins in intracellular genome segment recruitment is, however, less understood. Here we used our previously developed RVFV replicon particles, also referred as nonspreading RVFV (NSR) [27], to study the role of the glycoproteins in genome segment recruitment in more detail. NSR particles are phenotypically similar to wild-type virus, however they cannot spread autonomously because they lack the glycoprotein-encoding M genome segment.

**Fig 5:**
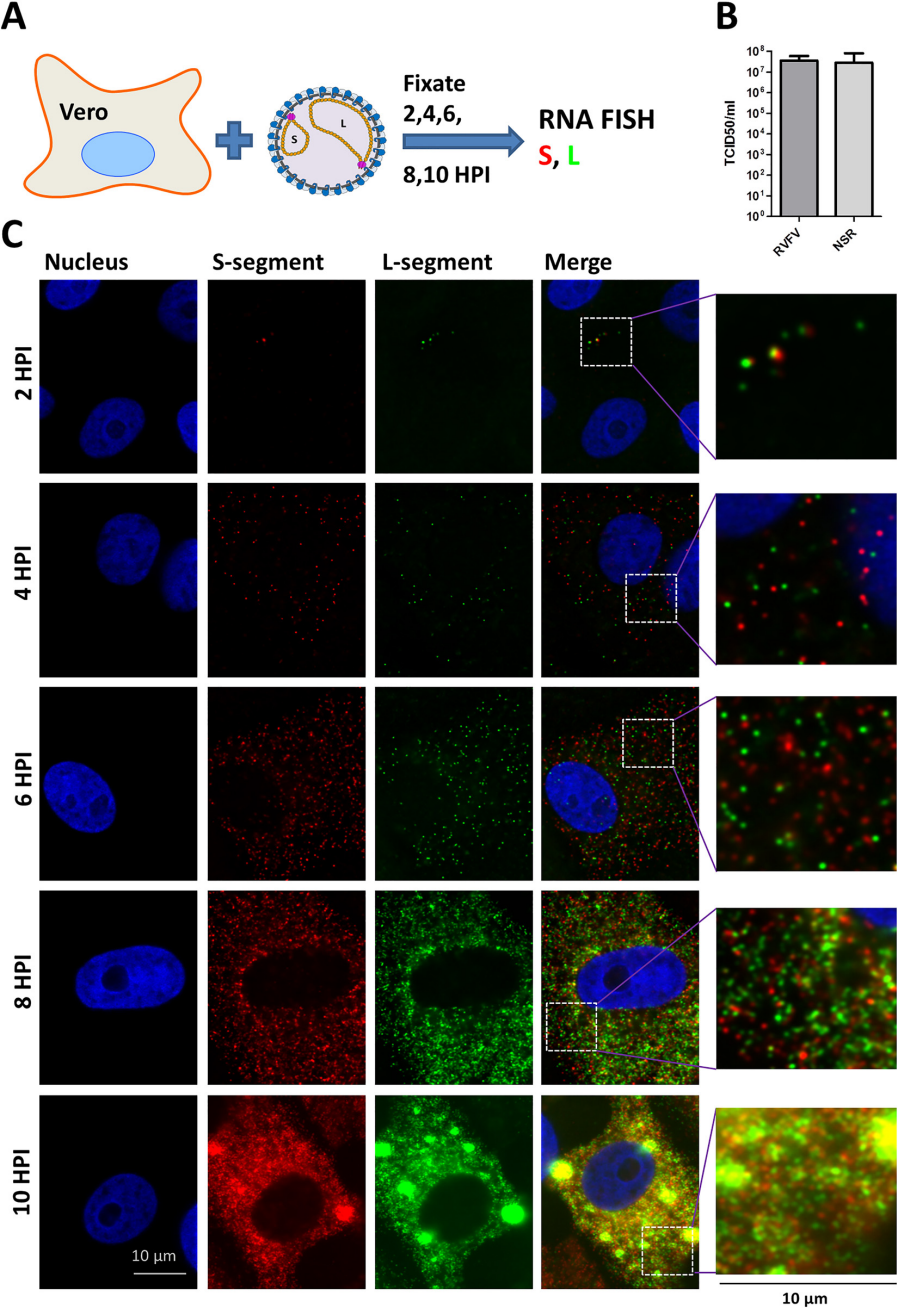
Single molecule vRNA FISH of NSR infected cells. (A) Schematic presentation of the experimental setup. (B) Maximal titers and SDs of wild-type RVFV and NSR stocks. (C) Spatio-temporal distribution of genome segments in NSR infected cells. Vero cells were infected at MOI 0.1 with NSR and cells were fixed at 2,4,6,8 and 10 hpi. Cells were subsequently probed against the S segment (N gene, red) using quasar 670 labelled probes and against the L-segment (polymerase gene, green) using quasar 570 labelled probes. Cell nuclei were visualized with dapi (blue). Images were taken using a wide-field microscope. Magnified images of the squared regions are shown at the right of each panel. The merge images show the spatial relationship between all the different channels.

Vero cells were infected with NSR and the spatio-temporal distributions of the S and L genome segments were determined by FISH (Fig 5). The results show that the total level of genome segments rapidly increased in time, similar as observed in RVFV infected cells (Fig 2). Importantly, no evidence of Golgi recruitment was observed at any time point (Fig 5). This suggests that in wild-type virus infected cells recruitment of genome segments is fully mediated by the glycoproteins, most likely mediated by the cytoplasmic tail of Gn, as was previously suggested by Piper and co-workers [19].

Remarkably, starting at 8 hpi, we consistently observed aggregates of genome segments in NSR-infected cells (Fig 5). The aggregates were randomly distributed and not associated with the Golgi. Probably, the absence of viral budding results in accumulation and subsequent aggregation of RNPs. In RVFV infected cells no such aggregates were found, not even at later time points.

### Genome segment replication and recruitment is out of balance in RVFV-4s infected cells

The NSR experiments suggested a major role for the RVFV glycoproteins, probably Gn, in genome segment recruitment. The RVFV glycoproteins Gn and Gc are normally produced from a glycoprotein precursor (GPC) protein that is proteolytically cleaved. Gn and Gc subsequently form heterodimers and mature at the endoplasmic reticulum (ER) and Golgi. Gn harbours a Golgi localization motif and Gc contains an ER retention signal [31]. We previously constructed RVFV-4s variants by splitting the M segment into two M-type segments encoding either the Gn or Gc protein [30]. We hypothesized that genome replication and recruitment is affected by changes in glycoprotein processing and genome organisation. To test this hypothesis we evaluated the spatio-temporal distribution of genome segments in RVFV-4s infected cells by FISH.

**Fig 6:**
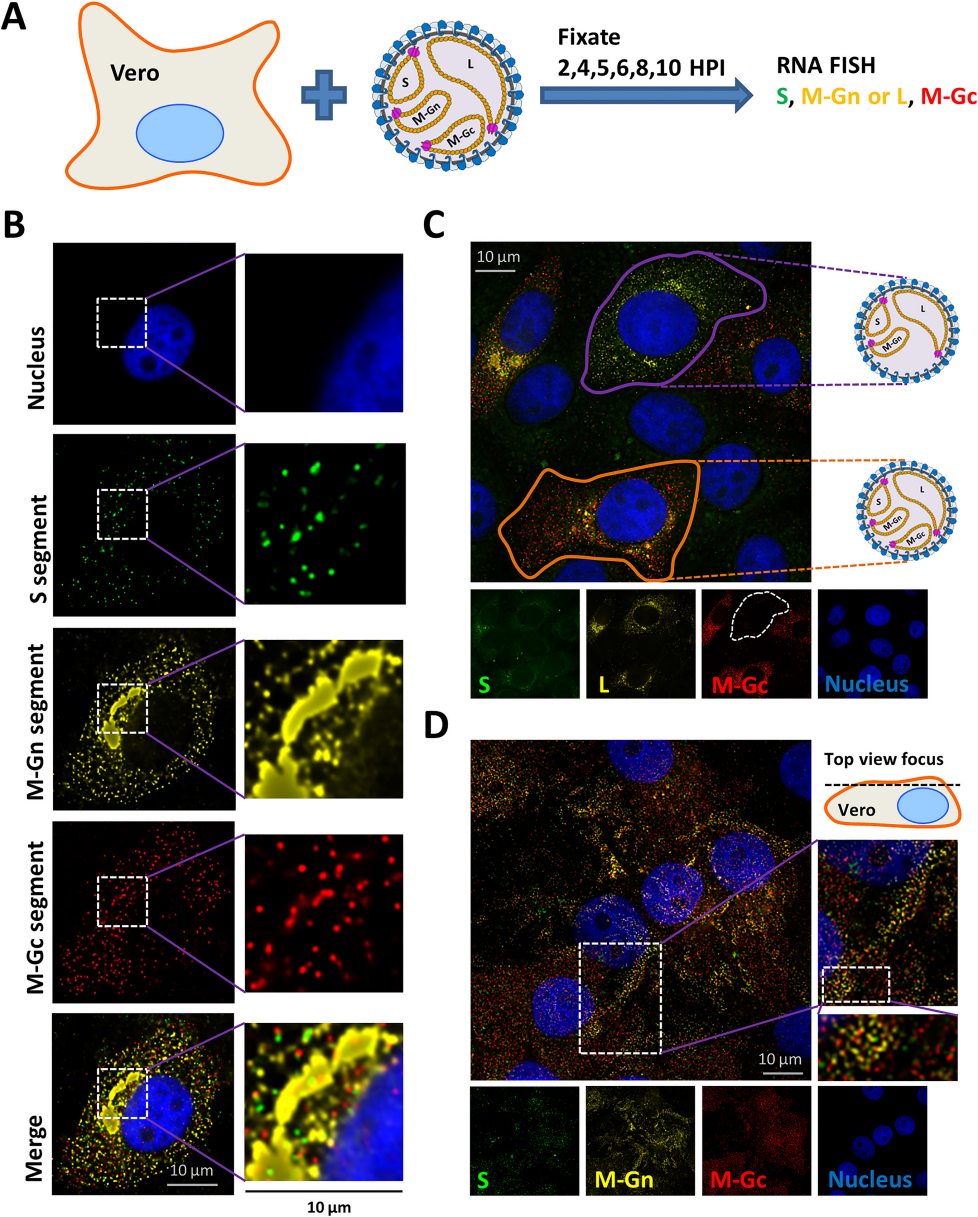
Single molecule vRNA FISH of RVFV-4s infected cells. (A) Schematic presentation of the experimental setup. Vero cells were infected at MOI 0.1 with RVFV-4s and cells were fixed at 6 (B,C) and 10 (D) hpi. Cells were subsequently probed against (B,D) the S segment (N gene, green) using fluorescein labelled probes, against the M-Gn segment (Gn gene, yellow) using quasar 570 labelled probes and against the M-Gc segment (Gc gene, red) using quasar 670 labelled probes or (C) against the S segment (N gene, green) using fluorescein labelled probes, against the L segment (polymerase gene, yellow) using quasar 570 labelled probes and against the M-Gc segment (Gc gene, red) using quasar 670 labelled probes. Cell nuclei were visualized with dapi (blue). Images were taken using a wide-field microscope. Magnified images of the squared regions are highlighted with white squares. The merged images show the spatial relationship between all the different channels.

Vero cells were infected with RVFV-4s and hybridized with probes complementary to the S, M-Gn, M-Gc and L genome segments. The results show that the M-Gn segment is replicated more efficiently compared to the M-Gc segment (Fig 2I). Moreover, recruitment of the M-Gn segment to the Golgi was much more efficient compared to recruitment of the other segments (Fig 6B). Recruitment of M-Gn was also more efficient compared to recruitment of the wild-type M-segment in RVFV infected cells (Figs 1 and 6B).

Since RVFV-4s is able to spread after infection at low MOI (< 0.001) a significant population of particles in a virus stock is expected to contain all four genome segments. Interestingly, the FISH experiments at 6 hpi revealed that various infected cells (up to 40%) did not show evidence of M-Gc replication (Fig 6C). Most likely these cells were originally infected with virions containing the S, M-Gn and L genome segments but lacking the M-Gc segment. The number of M-Gc lacking virions correlates very well with the reduced replication of the M-Gc segment (Fig 2I) and, like for wild-type virus, confirms that during particle assembly no quality control mechanisms are present that ensure packaging of all different segments, including M-Gc, into a single particle.

Another interesting observation in RVFV-4s infected cells was the reduced replication of the S segment. Most likely there is increased competition for polymerase molecules in RVFV-4s infected cells (4 instead of 3 segments) resulting in reduced replication of segments with a relative low affinity for the polymerase. Differences in polymerase affinity have already been shown at the transcription level [13].

A final characteristic of RVFV-4s infected cells was the presence of higher densities of genome segments near the plasma membrane later on in infection (Fig 6D), suggesting that in RVFV-4s infected cells, various Gn molecules move to the plasma membrane and bind genome segments during transit. The ability of Gn to move to the plasma membrane, especially in the absence of Gc is well known [22,31]. Whether RVFV-4s is able to bud at the plasma membrane awaits further study.

Altogether, the overall unbalance in genome segment replication, the enhanced Golgi recruitment of the M-Gn segment and the increased number of particles lacking one or more genome segments explain, at least partly, the observed attenuated phenotype of RVFV-4s [30].

### Rescue of a RVFV variant with a codon shuffled M-segment

**Fig 7:**
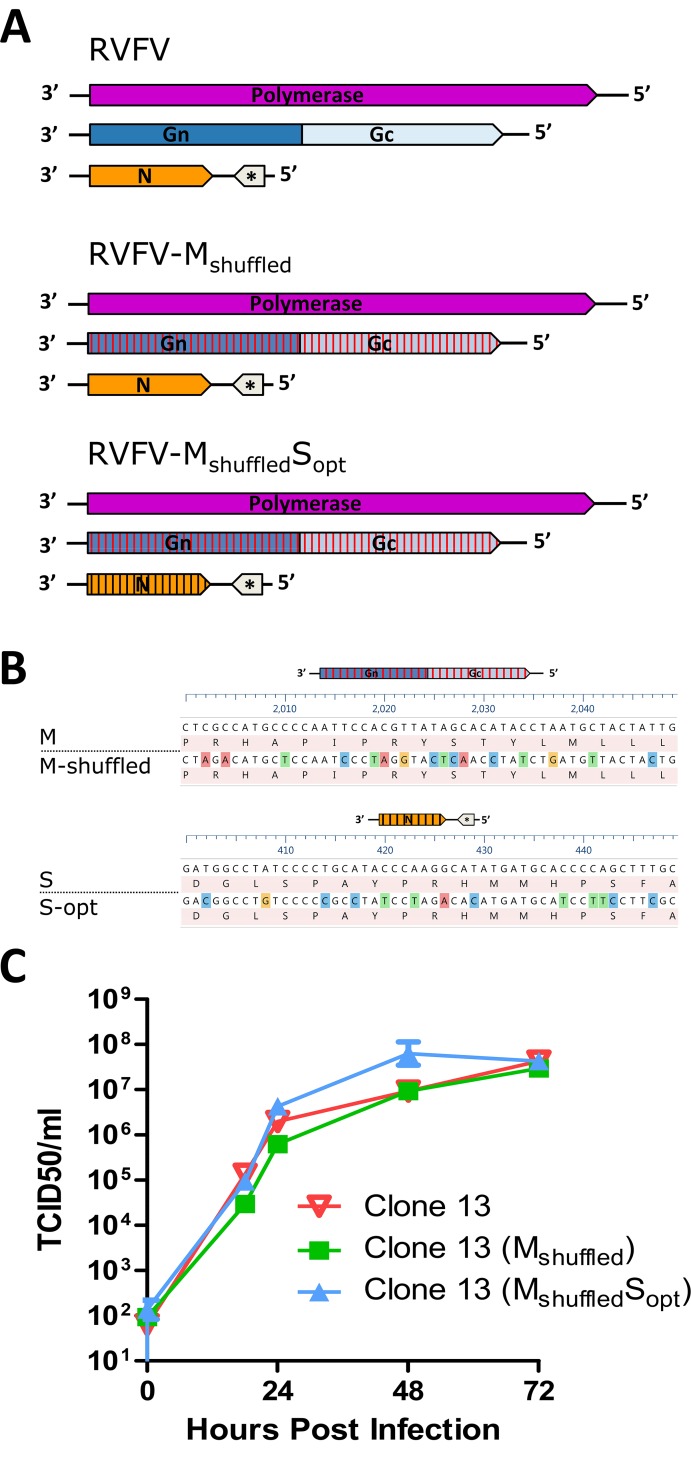
Growth of codon shuffled RVFV variants. (A) Schematic presentation of the viruses with shuffled or codon-optimized genes. (B) Part of the shuffled M segment and codon-optimized N gene sequence. (C) Growth curve of the indicated viruses in Vero cells infected at MOI 0.01. Supernatants were harvested at different time points and titrated on Vero cells.

Although the experiments thus far show that a supramolecular complex, consisting of an S, M and L genome segment is not formed in the cytoplasm, we cannot yet rule out the possibility that a supramolecular RNP complex is formed at the virion assembly site. During the influenza infection cycle, the formation of a supramolecular RNP complex is based on RNA-RNA interactions between the different segments and this process is believed to trigger viral budding [35,36]. To obtain additional information about the putative formation of a supramolecular RNP complex during the RVFV infection cycle we tried to rescue a RVFV variant with a codon shuffled M-segment (Fig 7 and S2 Fig). Codon shuffling changes the genomic RNA sequence but does not affect the protein sequence and has limited effects on protein expression. When RNA-RNA interactions exist between the S, M and L RNPs, a virus with a codon shuffled M segment is expected to grow less efficiently.

Interestingly, rescue of the RVFV variant with a codon shuffled M-segment, referred as RVFV-M_shuffled_, was successful. Moreover, we additionally rescued a RVFV variant with a shuffled M-segment and an optimized S segment, referred as RVFV-M_shuffled_S_opt_ (Figs 7 and S3). Both variants were able to grow with similar kinetics and to similar titers in Vero cells compared to the parental RVFV strain (Fig 7). The efficient growth of these variants further suggests that the formation of a supramolecular RNP complex does not drive the production of infectious RVFV virions.

### Heterogeneity in segment content virions

**Fig 8:**
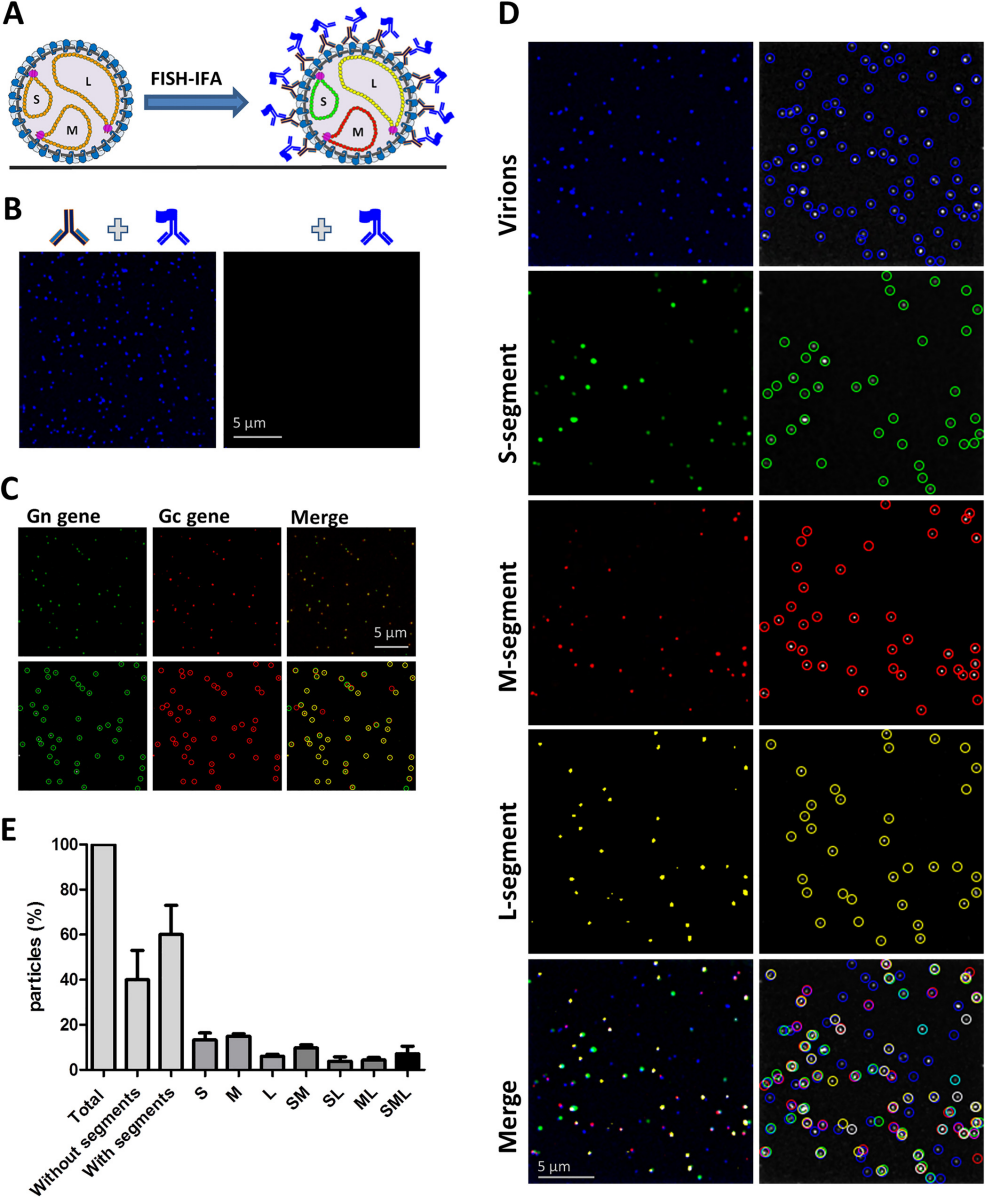
Genome segment composition of immobilized virions. (A) Schematic presentation of the experimental setup. (B) Control experiment to validate the visualization of immobilized RVFV virions and their genome segments. Immobilized virions were incubated in the presence (left image) or absence (right image) of the 4-39-cc mAb targeting the Gn glycoprotein followed by incubation with a DyLight 350 labelled conjugate. (C) Validation of the ability to determine segment colocalization inside immobilized virions. Immobilized RVFV virions were hybridized with a quasar 570 labelled Gn gene-specific probe set (green) and a quasar 670 labelled Gc gene-specific probe set (red). Since the Gn and Gc coding regions are both present on the M-genome segment, Gn and Gc gene-specific spots should show a high level of colocalization (in yellow). Colocalization percentages were on average 80% (D) Immobilized RVFV virions were hybridized with S segment specific probe sets (N gene, fluorescein, green), M segment specific probes sets (Gn and Gc, quasar 670, red) and L segment specific probe sets (polymerase, quasar 570, yellow) and incubated with the 4-39-cc Gn specific mAb in combination with the DyLight 350 labelled conjugate (blue). In each channel, spots were subsequently detected with the ComDet plugin of ImageJ and merged images of the four different channels are presented. (E) Quantification of the different genome compositions inside virions. About 800 virions were analysed for their genome content using the ComDet plugin of ImageJ.

Altogether, the presented results suggest that RVFV genome packaging is a non-selective process. To obtain additional evidence for this conclusion we evaluated the genome segment content of mature virions. Virions in wild-type virus stocks (produced on Vero cells) were immobilized on coverslips and incubated with antibodies targeting the Gn glycoprotein and probe sets recognising the S, M and L genome segments as described in the M&M section. After confirming specificity and the ability to determine colocalization with this assay (Fig 8B and 8C) the genome content of >800 virions was determined. As expected, the results revealed a high level of heterogeneity in genome composition. Virions were observed that did not comprise any genome segment (about 40%) as well as virions with only one or two segment types (Fig 8D and 8E). About 1 out of 10 virions showed evidence for the presence of all three different segments. The relatively low abundance of virions containing all the different segments is in full agreement with the FISH data obtained with infected cells and confirms the non-selective nature of RVFV genome packaging.

## Discussion

Although genome packaging of viruses with segmented genomes has intrigued researchers for decades, we are only just beginning to understand the molecular processes involved. In the field of segmented negative-strand RNA viruses, most knowledge resulted from studies with influenza virus. In the latest influenza model, genome packaging is proposed to be a highly selective process based on the formation of a supramolecular RNP complex [33–35,37,38]. From an evolutionary perspective, a selective genome packaging process for an 8-segmented virus is easily understood. If not selective, the influenza virus would need to produce about 400 particles to generate 1 particle that contains each of the 8 genome segments, which is rather inefficient. For bunyaviruses, which only have to package 3 segments, the evolutionary pressure to selectively incorporate genome segments during virion assembly is much lower. With this study, we provide evidence that RVFV uses a non-selective genome packaging strategy.

At the beginning of this study, limited knowledge was available about the molecular mechanisms involved in RVFV genome replication, recruitment and packaging. Moreover, as explained in the introduction section, some results pointed towards a highly selective genome packaging strategy whereas others were compatible with a non-selective packaging process. In the current study, we investigated the molecular mechanisms involved in RVFV genome packaging by combining new tools such as replicon particles, four-segmented- and codon-shuffled viruses with state-of-the-art single molecule RNA-FISH. The absence of colocalization of RNPs in the cytoplasm (Fig 4), the similar to wild-type growth of codon shuffled variants (Fig 7), the efficient production of replicon particles (Fig 5B), the observed heterogeneity in intracellular segment replication among infected cells (Fig 3 and S2 Fig) and the heterogeneity in segment composition of mature virions (Fig 8) demonstrate that the non-selective genome packaging model is the most plausible model to date. The non-selective genome packaging model is in full agreement with the ability to construct a wide variety of RVFV variants without the need to conserve coding sequences and RNA structures [26,29,30].

**Fig 9:**
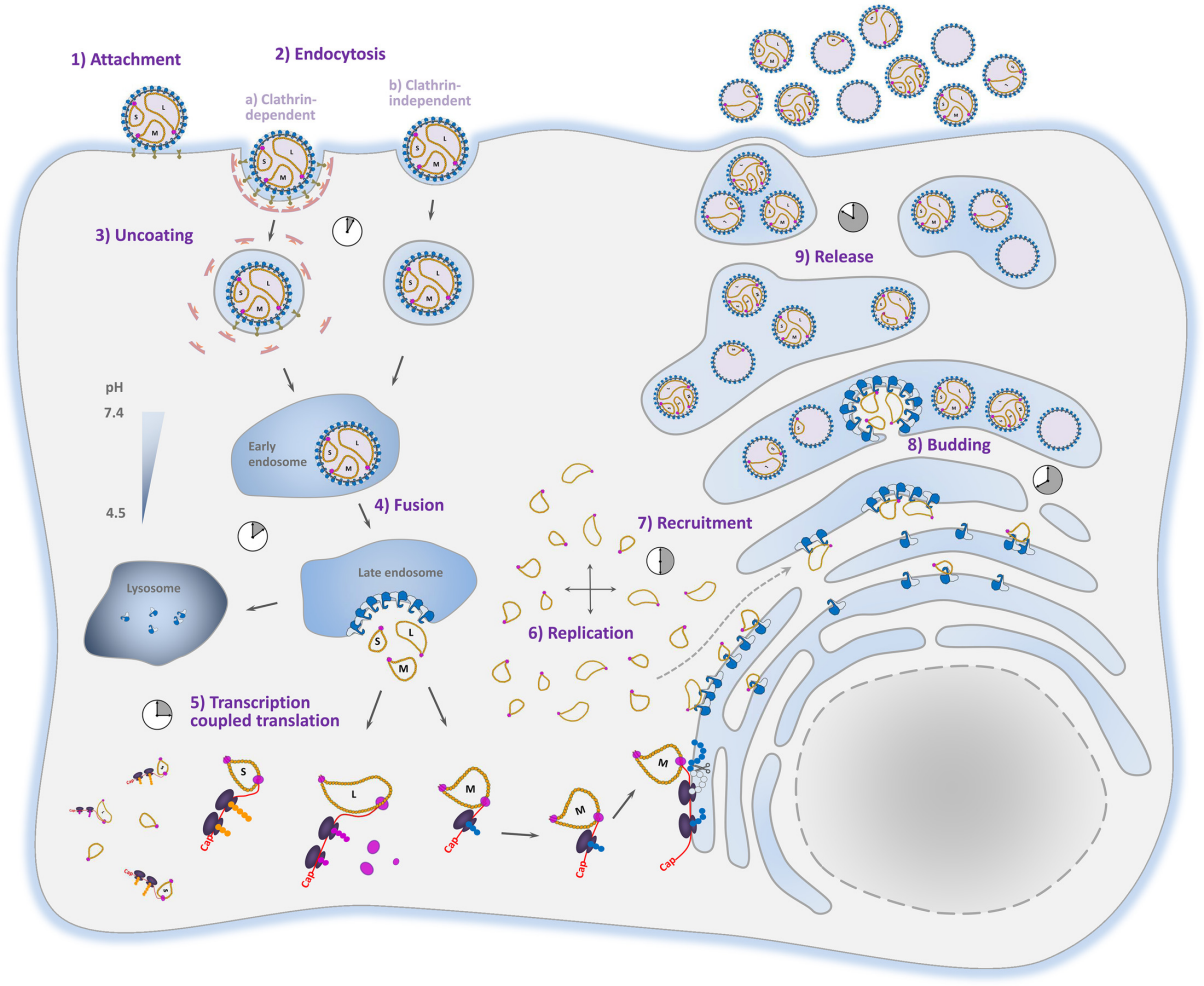
Schematic presentation of the RVFV replication cycle. 1) Upon virion attachment, the particles are endocytosed by clathrin-dependent (a) or independent (b) endocytosis. After acidification of the endosome, Gn and Gc undergo conformational changes resulting in fusion of the viral and endosomal membranes and subsequent release of the RNPs into the cytosol. Near the fusion site, the RNPs are used as templates for transcription and replication (5,6). L and S segment-encoded mRNAs are translated by free ribosomes whereas M segment encoded mRNA is translated by membrane bound ribosomes at the ER. Newly formed RNPs (4–6 hpi) migrate to random sites in the cytoplasm initiating additional rounds of replication followed by glycoprotein mediated recruitment to the Golgi (7). Glycoprotein heterodimers or higher-order glycoprotein structures are expected to bind RNPs via interaction of the N protein with the cytoplasmic tail of Gn. Finally, RNPs accumulate at the Golgi and virions are formed by budding into the Golgi lumen (8). Mature virus particles, containing zero to three, or perhaps even more genome segments, are released from the cell via exocytosis (9).

We here demonstrate that replication of RVFV genome segments starts locally, probably near the site of fusion of the virion with the endosome, and subsequently (within 4–6 h) continues to proceed throughout the cytoplasm. After the replication phase, genome segments are recruited to the Golgi. Recruitment is probably mediated by interactions of the nucleocapsid protein, which covers the viral RNA, with the cytoplasmic tail of Gn [19,22,24,25]. After recruitment, a very heterogeneous population of virions, containing various amounts and types of genome segments, buds into the Golgi lumen. Virions with at least one S, M and L RNP will be able to produce progeny virions upon infection. Alternatively, co-infection with complementing particles may result in productive infection. Interestingly, virions containing antigenomic-sense RNPs may also contribute to the RVFV infection cycle [29,39]. In Fig 9, a schematic presentation of the RVFV infection cycle, according to the newly obtained insights, is provided.

Although our results suggest that a supramolecular RNP complex is not formed, or at least does not play a critical role in the RVFV replication cycle, we cannot exclude that some degree of selectivity exists, as has been previously suggested [17,18]. If some degree of selection indeed occurs, our results obtained with codon-shuffled variants suggest that this selection is mediated by the UTRs.

A major finding in the RVFV-4s infected cells was the difference in replication efficiency of the M-Gn versus M-Gc segment. The difference in replication is not explained by large differences in segment size (2319 nt versus 1869 nt) or differences in UTR sequence, since these are identical. An explanation might be that the NSm coding region, which is present in the M-Gn segment but absent from the M-Gc segment, contains a yet unknown cis-acting replication element. At first glance, the efficient replication of codon-shuffled variants seems to contradict this hypothesis. However, a short stretch of nucleotides downstream of the 5’ UTR and a short stretch of nucleotides upstream of the GnGc open reading frame were maintained in these viruses (S3 Fig) to preserve efficient translation. These sequences are not present in the M-Gc segment and could be involved in replication. Future research will determine if these sequences indeed contain cis-acting replication signals.

Another very consistent finding throughout the experiments was the enhanced Golgi recruitment of the M segment compared to the S and L segments in wild-type virus infected cells and the enhanced recruitment of the M-Gn segment in RVFV-4s infected cells. The enhanced recruitment was calculated by dividing the cytoplasmic segment ratios before (4 hpi) and after Golgi localization (8 hpi). The percentage of cytoplasmic M-segments decreased with 16% in wild-type virus infected cells and the percentage of M-Gn segments decreased with 11% compared to the other segments in RVFV-4s infected cells. The enhanced recruitment of Gn encoding segments can be explained by the coupled transcription and translation in bunyaviruses. Specifically, we propose the following sequence of events: transcription of the M segment is initiated in the cytoplasm, followed by translation of the Gn signal sequence by free ribosomes. A complex of M genome segments, mRNA transcribed from this segment and ribosomes is then translocated to the ER and subsequently to the Golgi compartment to continue membrane-associated translation of M segment mRNAs.

Although the current study provides evidence for a non-selective genome packaging process during RVFV virion assembly, we do not think these results can be extrapolated to all bunyaviruses. Whereas RVFV RNPs are expected to bind to the cytoplasmic tail of the Gn protein via the N protein, for other bunyaviruses, such as Crimean Congo hemorrhagic fever virus (CCHFV), evidence was provided that the viral RNA directly interacts with the cytoplasmic tail of the Gn protein [40]. This N-independent interaction might be segment specific and could facilitate a more selective packaging process. The latter could also explain the lower particle to PFU ratio of CCHF compared to RVFV [41].

In summary, this study suggests that RVFV genome packaging is a non-selective process and does not involve the formation of a supramolecular viral RNA complex.

## Materials and Methods

### Viruses and cells

The RVFV strain Clone 13 [42] was kindly provided by Dr. Michèle Bouloy (Institut Pasteur, France). RVFV-4s, RVFV-M_shuffled_ and RVFV-M_shuffled_S_opt_ were constructed using reverse genetics. Sequences were based on the published Clone 13 genome (Accession: DQ375417.1, DQ380213.1, DQ380182.1). Working stocks were obtained by low MOI (0.01) infections of Vero E6 cells (ATCC CRL-1586) grown in Eagle's Minimum Essential Medium (EMEM) supplemented with 5% FBS, 1% non-essential amino acids, 1% L-glutamine and 1% antibiotic/antimycotic. RVFV replicon (NSR) stocks were obtained by transfection of replicon cells, which stably maintain replicating S and L genome segments with an expression plasmid expressing the RVFV glycoproteins as described previously [27].

### Plasmids

RVFV sequences, flanked by a minimal T7 promoter and a hepatitis delta virus ribozyme sequence, were synthesized by the GenScript Corporation (New Jersey, USA) and cloned into pUC57 plasmids. RVFV-4s M-type plasmids were designed (Clone 13 sequence based), as previously described, to contain half of the GPC gene, either encoding (NSm)Gn or Gc (segmented at the tyrosine-675 codon of the GPC) [30]. The RVFV-M_shuffled_ segment was designed by shuffling of the NSmGnGc gene resulting in 77% homology. The RVFV-S_opt_ plasmid contains a codon-optimized N gene for optimal expression in mammalian cells. NSmGnGc shuffled and N optimized sequences are presented in S2 Fig and S3 Fig respectively.

### Reverse genetics

RVFV-4s, RVFV-M_shuffled_ and RVFV-M_shuffled_S_opt_ were rescued using a three (or four for RVFV-4s) plasmid system. Briefly, BSR-T7/5 cells [43] (previously kindly provided by Prof. Karl-Klaus Conzelmann) were seeded in T75 flasks (2,500,000 cells/flask) in GMEM containing 5% FBS and after overnight incubation medium was replaced with Opti-MEM. Cells were transfected with a total of 20 μg pUC57 transcription plasmids per flask using TransIT transfection reagents according the manufacturers’ instructions (Mirus, MAD). Three to five days post transfection, supernatants were collected and used to infect Vero E6 cells.

### RNA-FISH

All RNA-FISH assays were performed according the Stellaris FISH method originally developed by Ray, Femino and co-workers [44,45].

For the RNA-FISH cell assays Vero E6 cells (15,000 cells/well) were seeded on CultureWell 16 Chambered Coverglass (Grace Biolabs). After overnight incubation, cells were incubated with the indicated viruses for 1 h (MOI 0.1–0.01) and at the indicated time points infected cells were fixed for 10 min with fixation buffer (75% methanol, 25% glacial acetic acid). Cells were subsequently washed with PBS (5 min) and pre-hybridization buffer (5 min) consisting of 10% formamide and 2 mM vanadyl ribonucleoside complex (VRC) in 2x concentrated SSC. Subsequently, cells were probed overnight (18 h) at 37°C in hybridization buffer (10% formamide, 2 mM VRC, 10% w/v Dextran-Sulphate in 2 times SSC) with the indicated probe sets (S1 Table) at an end concentration of 125 nM. The probes were designed using the RNA FISH Probe Designer available online at www.biosearchtech.com and purchased from Biosearch Technologies Inc. (Petaluma, CA). After the hybridization, cells were extensively washed with pre-hybridization buffer and 2 times SSC. Cell nuclei were visualized using DAPI and prior imaging, cells were submerged in VectaShield mounting medium (H-1000, Vector Laboratories).

For the RNA-FISH virion assays, undiluted virus stocks were incubated for 3 h in the CultureWell 16 Chambered Coverglass wells at 37°C. The negatively charged glass binds virions relatively efficient. After bound virions were fixed and hybridized according the procedure described for cells, with the only exception that hybridization time was reduced to 4 h, virions were visualized with the RVFV-Gn specific monoclonal antibody 4-39-cc [46] in combination with a DyLight 350 labelled Rabbit anti-Mouse (H+L) conjugate (ThermoFisher Scientific). Immobilized virions were finally submerged in VectaShield prior imaging.

### Image acquisition and analysis

Images of infected cells and immobilized virions were obtained with an inverted fluorescence wide-field ZEISS Axioskop 40 microscope with appropriate filters and a 1.3 NA 100× oil objective in combination with an Axiocam MRm CCD camera. Raw cell images were subsequently deconvolved and analysed using Huygens deconvolution software (SVI, Hilversum, The Netherlands) in combination with the ImageJ program (National Institutes of Health, USA). Spots (individual vRNAs) were counted using the StarSearch algorithm http://rajlab.seas.upenn.edu/StarSearch/launch.html. Images of coverslip immobilized virions were analysed by ImageJ in combination with the ComDet plugin https://github.com/ekatrukha/ComDet/wiki.

## Supporting Information

S1 Fig Sensitivity and specificity of the probe sets. A) Uninfected Vero cells or (B) RVFV infected Vero cells were probed against the S segment (N gene) using fluorescein labelled probes (green), against the M segment (Gn and Gc gene) using quasar 670 labelled probes (red) and against the L-segment (polymerase gene) using quasar 570 labelled probes (blue). A representative example of a fluorescence intensity peak, characteristic of single molecules, is presented at the bottom of the image. C) Histogram of the fluorescence spots intensity of the unprocessed M-segment channel of the image presented in Fig 4. The spot intensity distribution displays an almost Gaussian distribution (red line), which is characteristic of single molecules.
(TIF)

S2 Fig Representative panel of images obtained after single molecule vRNA FISH of RVFV infected cells. Vero cells were infected with RVFV at MOI 0.1 and fixed at 7 hpi. Cells were subsequently probed against the S segment (N gene) using fluorescein labelled probes (green), against the M segment (Gn and Gc gene) using quasar 670 labelled probes (red) and against the L-segment (polymerase gene) using quasar 570 labelled probes (blue). Cell nuclei were visualized with dapi (cyan). Infected cells that did not show evidence of M-segment replication are indicated with a dashed line.
(TIF)

S3 Fig Sequence shuffled M segment. Alignment of the shuffled M segment sequence with the wild-type sequence.
(TIF)

S4 Fig Sequence optimized S segment. Alignment of the optimized S segment sequence with the wild-type sequence.
(TIF)

S1 Table FISH probe sequences. (DOCX)
