# Supplementary material for: Single-Molecule FISH Reveals Non-selective Packaging of Rift Valley Fever Virus Genome Segments
Source: PLoS Pathog. 2016 Aug 22;12(8):e1005800. doi: 10.1371/journal.ppat.1005800 (PMC4993503; doi:10.1371/journal.ppat.1005800)
Supplement: S1 Table — (DOCX) [file ppat.1005800.s005.docx]

| **Probe set** | **Sequence Name** | **Sequence** | **Label** |
| --- | --- | --- | --- |
| Polymerase Q570 | Polymerase_1 | gtcggatctatgttctcatt | Quasar 570 |
| Polymerase Q570 | Polymerase_2 | gagatgaaagtacggggctc | Quasar 570 |
| Polymerase Q570 | Polymerase_3 | agacatgggggtcacaaaca | Quasar 570 |
| Polymerase Q570 | Polymerase_4 | ttatggagttgggatctgga | Quasar 570 |
| Polymerase Q570 | Polymerase_5 | gaacagtgggtggattcatc | Quasar 570 |
| Polymerase Q570 | Polymerase_6 | ttgggatccttcagagattt | Quasar 570 |
| Polymerase Q570 | Polymerase_7 | tctgttctgcatactacagg | Quasar 570 |
| Polymerase Q570 | Polymerase_8 | cagctgaaatcctcaagcac | Quasar 570 |
| Polymerase Q570 | Polymerase_9 | aagcaactctagctcacact | Quasar 570 |
| Polymerase Q570 | Polymerase_10 | tcgggactcagaagagtagg | Quasar 570 |
| Polymerase Q570 | Polymerase_11 | tattcctattcccgaacatt | Quasar 570 |
| Polymerase Q570 | Polymerase_12 | gttgtgtcatcattgtcttc | Quasar 570 |
| Polymerase Q570 | Polymerase_13 | tgcgcattgcagagaaagtc | Quasar 570 |
| Polymerase Q570 | Polymerase_14 | gagaccgtttgaacatacca | Quasar 570 |
| Polymerase Q570 | Polymerase_15 | gatgttaccatcccagaaac | Quasar 570 |
| Polymerase Q570 | Polymerase_16 | tttcttcctgcttgacaatc | Quasar 570 |
| Polymerase Q570 | Polymerase_17 | ccttagctgcaatgattcag | Quasar 570 |
| Polymerase Q570 | Polymerase_18 | atgttagaccgacgatcagg | Quasar 570 |
| Polymerase Q570 | Polymerase_19 | gaattttatttccacaccca | Quasar 570 |
| Polymerase Q570 | Polymerase_20 | agtagcatgctaatcagctt | Quasar 570 |
| Polymerase Q570 | Polymerase_21 | ttgctcctgagatgagcaaa | Quasar 570 |
| Polymerase Q570 | Polymerase_22 | cggtccttgtcctttaaaat | Quasar 570 |
| Polymerase Q570 | Polymerase_23 | cttcctcattattacacacc | Quasar 570 |
| Polymerase Q570 | Polymerase_24 | tggtcatcgagagcttgata | Quasar 570 |
| Polymerase Q570 | Polymerase_25 | attagggctcggaagcaatg | Quasar 570 |
| Polymerase Q570 | Polymerase_26 | tagggaagttctttgcttct | Quasar 570 |
| Polymerase Q570 | Polymerase_27 | tcaatcggtggtggagacaa | Quasar 570 |
| Polymerase Q570 | Polymerase_28 | ccagaagtttgaggattgca | Quasar 570 |
| Polymerase Q570 | Polymerase_29 | acttaaggccacaagcaact | Quasar 570 |
| Polymerase Q570 | Polymerase_30 | atcatgctaagctggtctta | Quasar 570 |
| Polymerase Q570 | Polymerase_31 | ctgcttatgcagaactatcc | Quasar 570 |
| Polymerase Q570 | Polymerase_32 | agagctgcaggtttacttat | Quasar 570 |
| Polymerase Q570 | Polymerase_33 | ttccaagctggaactttgtt | Quasar 570 |
| Polymerase Q570 | Polymerase_34 | gcgtttcgttaatggaatcc | Quasar 570 |
| Polymerase Q570 | Polymerase_35 | gtcttttgcaattaagcgct | Quasar 570 |
| Polymerase Q570 | Polymerase_36 | tcagccatccttatcagaaa | Quasar 570 |
| Polymerase Q570 | Polymerase_37 | tagtgacttgtgggcaacag | Quasar 570 |
| Polymerase Q570 | Polymerase_38 | cttctctccagatcatgatg | Quasar 570 |
| Polymerase Q570 | Polymerase_39 | caacctgtggagagaagtgg | Quasar 570 |
| Polymerase Q570 | Polymerase_40 | gtgagcagatgcctcataaa | Quasar 570 |
| Polymerase Q570 | Polymerase_41 | ccgattcagagtacatcacg | Quasar 570 |
| Polymerase Q570 | Polymerase_42 | gtcagtcacagaatctgtgt | Quasar 570 |
| Polymerase Q570 | Polymerase_43 | gtctcctccatccaaataaa | Quasar 570 |
| Polymerase Q570 | Polymerase_44 | atgagctaaggaccttgttc | Quasar 570 |
| Polymerase Q570 | Polymerase_45 | aggactctgaagcagaggag | Quasar 570 |
| Polymerase Q570 | Polymerase_46 | ggatgaaagaggtgcattcc | Quasar 570 |
| Polymerase Q570 | Polymerase_47 | tgacgggtttgatcatctga | Quasar 570 |
| Polymerase Q570 | Polymerase_48 | gactggttttgttagagtgc | Quasar 570 |
|  |  |  |  |
| **Probe set** | **Sequence Name** | **Sequence** | **Label** |
| NSmGn Q570 | NSmGn_1 | taggatggatggaaggaggc | Quasar 570 |
| NSmGn Q570 | NSmGn_2 | gaagatggttgccagagtag | Quasar 570 |
| NSmGn Q570 | NSmGn_3 | actgttttcatcagatgggt | Quasar 570 |
| NSmGn Q570 | NSmGn_4 | aggaaagttctggatccact | Quasar 570 |
| NSmGn Q570 | NSmGn_5 | tcaagtgcctaaagattgcc | Quasar 570 |
| NSmGn Q570 | NSmGn_6 | cgcaataatttgtttggcca | Quasar 570 |
| NSmGn Q570 | NSmGn_7 | gttgtgttcgtatttagctc | Quasar 570 |
| NSmGn Q570 | NSmGn_8 | tgctcatggcctgataaatt | Quasar 570 |
| NSmGn Q570 | NSmGn_9 | aaaatagtagctcactgccc | Quasar 570 |
| NSmGn Q570 | NSmGn_10 | gttcacatggcacatgatga | Quasar 570 |
| NSmGn Q570 | NSmGn_11 | tacactcaagtatccaggga | Quasar 570 |
| NSmGn Q570 | NSmGn_12 | agatatcatctgcagttgct | Quasar 570 |
| NSmGn Q570 | NSmGn_13 | aaatgtgagcctcacggatt | Quasar 570 |
| NSmGn Q570 | NSmGn_14 | tgagagggtggttgtgaaga | Quasar 570 |
| NSmGn Q570 | NSmGn_15 | acagatacaagtatccgggg | Quasar 570 |
| NSmGn Q570 | NSmGn_16 | tgttcacatgctaatgggtc | Quasar 570 |
| NSmGn Q570 | NSmGn_17 | tgagtgcactgctcaatacg | Quasar 570 |
| NSmGn Q570 | NSmGn_18 | agattggaggacatggtagc | Quasar 570 |
| NSmGn Q570 | NSmGn_19 | ccaagagggagctcaaaagc | Quasar 570 |
| NSmGn Q570 | NSmGn_20 | ggaacaatggactctggtca | Quasar 570 |
| NSmGn Q570 | NSmGn_21 | tttgtctgcttcgagcataa | Quasar 570 |
| NSmGn Q570 | NSmGn_22 | agcttgacctcaagactgag | Quasar 570 |
| NSmGn Q570 | NSmGn_23 | aactcagcactgcacatgag | Quasar 570 |
| NSmGn Q570 | NSmGn_24 | atgaaaggggtctgcgaagt | Quasar 570 |
| NSmGn Q570 | NSmGn_25 | tcctcttcaggacttatttt | Quasar 570 |
| NSmGn Q570 | NSmGn_26 | tgacaggtgctagcttgaag | Quasar 570 |
| NSmGn Q570 | NSmGn_27 | aggactgcaatttttgcagg | Quasar 570 |
| NSmGn Q570 | NSmGn_28 | cctcctaaatatgagctcac | Quasar 570 |
| NSmGn Q570 | NSmGn_29 | cgggaaaatggcttcagtca | Quasar 570 |
| NSmGn Q570 | NSmGn_30 | cactgtccaaatgactacca | Quasar 570 |
| NSmGn Q570 | NSmGn_31 | tccctgcatgaaggagaaac | Quasar 570 |
| NSmGn Q570 | NSmGn_32 | atcattgcaaaggctgatcc | Quasar 570 |
| NSmGn Q570 | NSmGn_33 | aaccctactagaagcagttc | Quasar 570 |
| NSmGn Q570 | NSmGn_34 | gaaaagggaaaattccccct | Quasar 570 |
| NSmGn Q570 | NSmGn_35 | gcacaactacattgacggga | Quasar 570 |
| NSmGn Q570 | NSmGn_36 | tctcagaaacagaccaggga | Quasar 570 |
| NSmGn Q570 | NSmGn_37 | cctgttgtttttgctgaaga | Quasar 570 |
| NSmGn Q570 | NSmGn_38 | cttagcagtttttgctttgg | Quasar 570 |
| NSmGn Q570 | NSmGn_39 | gattgcaatgacagtccttc | Quasar 570 |
| NSmGn Q570 | NSmGn_40 | tagttcggtgtgagagacga | Quasar 570 |
| NSmGn Q570 | NSmGn_41 | taaagggtcctcctcacaaa | Quasar 570 |
| NSmGn Q570 | NSmGn_42 | atggtaaggatcctgaggat | Quasar 570 |
| NSmGn Q570 | NSmGn_43 | ggcttgaacaacatcacctg | Quasar 570 |
| NSmGn Q570 | NSmGn_44 | aaaagccatcattgctgctg | Quasar 570 |
| NSmGn Q570 | NSmGn_45 | gttccatatcaggcataagg | Quasar 570 |
| NSmGn Q570 | NSmGn_46 | tgaaggagcttgggattcac | Quasar 570 |
| NSmGn Q570 | NSmGn_47 | agagaagagacctgctttgg | Quasar 570 |
| NSmGn Q570 | NSmGn_48 | tagagtgtctctaagttcca | Quasar 570 |
|  |  |  |  |
| **Probe set** | **Sequence Name** | **Sequence** | **Label** |
| NSmGn Q670 | NSmGn-2_1 | taggatggatggaaggaggc | Quasar 670 |
| NSmGn Q670 | NSmGn-2_2 | gaagatggttgccagagtag | Quasar 670 |
| NSmGn Q670 | NSmGn-2_3 | actgttttcatcagatgggt | Quasar 670 |
| NSmGn Q670 | NSmGn-2_4 | aggaaagttctggatccact | Quasar 670 |
| NSmGn Q670 | NSmGn-2_5 | tcaagtgcctaaagattgcc | Quasar 670 |
| NSmGn Q670 | NSmGn-2_6 | cgcaataatttgtttggcca | Quasar 670 |
| NSmGn Q670 | NSmGn-2_7 | gttgtgttcgtatttagctc | Quasar 670 |
| NSmGn Q670 | NSmGn-2_8 | tgctcatggcctgataaatt | Quasar 670 |
| NSmGn Q670 | NSmGn-2_9 | aaaatagtagctcactgccc | Quasar 670 |
| NSmGn Q670 | NSmGn-2_10 | gttcacatggcacatgatga | Quasar 670 |
| NSmGn Q670 | NSmGn-2_11 | tacactcaagtatccaggga | Quasar 670 |
| NSmGn Q670 | NSmGn-2_12 | agatatcatctgcagttgct | Quasar 670 |
| NSmGn Q670 | NSmGn-2_13 | aaatgtgagcctcacggatt | Quasar 670 |
| NSmGn Q670 | NSmGn-2_14 | tgagagggtggttgtgaaga | Quasar 670 |
| NSmGn Q670 | NSmGn-2_15 | acagatacaagtatccgggg | Quasar 670 |
| NSmGn Q670 | NSmGn-2_16 | tgttcacatgctaatgggtc | Quasar 670 |
| NSmGn Q670 | NSmGn-2_17 | tgagtgcactgctcaatacg | Quasar 670 |
| NSmGn Q670 | NSmGn-2_18 | agattggaggacatggtagc | Quasar 670 |
| NSmGn Q670 | NSmGn-2_19 | ccaagagggagctcaaaagc | Quasar 670 |
| NSmGn Q670 | NSmGn-2_20 | ggaacaatggactctggtca | Quasar 670 |
| NSmGn Q670 | NSmGn-2_21 | tttgtctgcttcgagcataa | Quasar 670 |
| NSmGn Q670 | NSmGn-2_22 | agcttgacctcaagactgag | Quasar 670 |
| NSmGn Q670 | NSmGn-2_23 | aactcagcactgcacatgag | Quasar 670 |
| NSmGn Q670 | NSmGn-2_24 | atgaaaggggtctgcgaagt | Quasar 670 |
| NSmGn Q670 | NSmGn-2_25 | tcctcttcaggacttatttt | Quasar 670 |
| NSmGn Q670 | NSmGn-2_26 | tgacaggtgctagcttgaag | Quasar 670 |
| NSmGn Q670 | NSmGn-2_27 | aggactgcaatttttgcagg | Quasar 670 |
| NSmGn Q670 | NSmGn-2_28 | cctcctaaatatgagctcac | Quasar 670 |
| NSmGn Q670 | NSmGn-2_29 | cgggaaaatggcttcagtca | Quasar 670 |
| NSmGn Q670 | NSmGn-2_30 | cactgtccaaatgactacca | Quasar 670 |
| NSmGn Q670 | NSmGn-2_31 | tccctgcatgaaggagaaac | Quasar 670 |
| NSmGn Q670 | NSmGn-2_32 | atcattgcaaaggctgatcc | Quasar 670 |
| NSmGn Q670 | NSmGn-2_33 | aaccctactagaagcagttc | Quasar 670 |
| NSmGn Q670 | NSmGn-2_34 | gaaaagggaaaattccccct | Quasar 670 |
| NSmGn Q670 | NSmGn-2_35 | gcacaactacattgacggga | Quasar 670 |
| NSmGn Q670 | NSmGn-2_36 | tctcagaaacagaccaggga | Quasar 670 |
| NSmGn Q670 | NSmGn-2_37 | cctgttgtttttgctgaaga | Quasar 670 |
| NSmGn Q670 | NSmGn-2_38 | cttagcagtttttgctttgg | Quasar 670 |
| NSmGn Q670 | NSmGn-2_39 | gattgcaatgacagtccttc | Quasar 670 |
| NSmGn Q670 | NSmGn-2_40 | tagttcggtgtgagagacga | Quasar 670 |
| NSmGn Q670 | NSmGn-2_41 | taaagggtcctcctcacaaa | Quasar 670 |
| NSmGn Q670 | NSmGn-2_42 | atggtaaggatcctgaggat | Quasar 670 |
| NSmGn Q670 | NSmGn-2_43 | ggcttgaacaacatcacctg | Quasar 670 |
| NSmGn Q670 | NSmGn-2_44 | aaaagccatcattgctgctg | Quasar 670 |
| NSmGn Q670 | NSmGn-2_45 | gttccatatcaggcataagg | Quasar 670 |
| NSmGn Q670 | NSmGn-2_46 | tgaaggagcttgggattcac | Quasar 670 |
| NSmGn Q670 | NSmGn-2_47 | agagaagagacctgctttgg | Quasar 670 |
| NSmGn Q670 | NSmGn-2_48 | tagagtgtctctaagttcca | Quasar 670 |
|  |  |  |  |
| **Probe set** | **Sequence Name** | **Sequence** | **Label** |
| N Q670 | N_1 | cgtacaagacagcagcctaa | Quasar 670 |
| N Q670 | N_2 | agcagctgtcatggcagctg | Quasar 670 |
| N Q670 | N_3 | tggattccaatgggaagccg | Quasar 670 |
| N Q670 | N_4 | ttcttgaaagcttttgggct | Quasar 670 |
| N Q670 | N_5 | aagccatgagaagaggagag | Quasar 670 |
| N Q670 | N_6 | gcagcagtgaatagcaactt | Quasar 670 |
| N Q670 | N_7 | aacgttcacgcagccaatga | Quasar 670 |
| N Q670 | N_8 | gaacaaaggaggaggttgcc | Quasar 670 |
| N Q670 | N_9 | atcaacccaaacctccgagg | Quasar 670 |
| N Q670 | N_10 | tctgctgcagttctcccggg | Quasar 670 |
| N Q670 | N_11 | tattggatgctcactctctg | Quasar 670 |
| N Q670 | N_12 | ccagaagactatctaagggc | Quasar 670 |
| N Q670 | N_13 | tggcatggtggacccttctc | Quasar 670 |
| N Q670 | N_14 | atatgatgcaccccagcttt | Quasar 670 |
| N Q670 | N_15 | ctatcccctgcatacccaag | Quasar 670 |
| N Q670 | N_16 | cactgggaccaccatggatg | Quasar 670 |
| N Q670 | N_17 | tcttgagtgagtggcttcct | Quasar 670 |
| N Q670 | N_18 | ctggacttgccaggctttgg | Quasar 670 |
| N Q670 | N_19 | acgagttgctgctgccctgg | Quasar 670 |
| N Q670 | N_20 | cccgggatgagttgactcta | Quasar 670 |
| N Q670 | N_21 | aagctaaaggaggggaatcc | Quasar 670 |
| N Q670 | N_22 | tggaggctctcatcaacaag | Quasar 670 |
| N Q670 | N_23 | gtcgaaagaaggcaaagcaa | Quasar 670 |
| N Q670 | N_24 | cccggaggatgatgatgaaa | Quasar 670 |
| N Q670 | N_25 | gctctgactcgtggcaacaa | Quasar 670 |
| N Q670 | N_26 | tgccaagaaaatgattgttc | Quasar 670 |
| N Q670 | N_27 | gtggggctgactgggagaag | Quasar 670 |
| N Q670 | N_28 | atcgaactcttaaagcagta | Quasar 670 |
| N Q670 | N_29 | aaggatttgatgcccgtagg | Quasar 670 |
| N Q670 | N_30 | tgggtccgagagtttgctta | Quasar 670 |
| N Q670 | N_31 | gtggaccgcaatgagattga | Quasar 670 |
| N Q670 | N_32 | gatccagtttgctgctcaag | Quasar 670 |
| N Q670 | N_33 | tggacaactatcaagagctt | Quasar 670 |
|  |  |  |  |
| **Probe set** | **Sequence Name** | **Sequence** | **Label** |
| N Fluorescein | N-2_1 | cgtacaagacagcagcctaa | C3-Fluorescein |
| N Fluorescein | N-2_2 | agcagctgtcatggcagctg | C3-Fluorescein |
| N Fluorescein | N-2_3 | tggattccaatgggaagccg | C3-Fluorescein |
| N Fluorescein | N-2_4 | ttcttgaaagcttttgggct | C3-Fluorescein |
| N Fluorescein | N-2_5 | aagccatgagaagaggagag | C3-Fluorescein |
| N Fluorescein | N-2_6 | gcagcagtgaatagcaactt | C3-Fluorescein |
| N Fluorescein | N-2_7 | aacgttcacgcagccaatga | C3-Fluorescein |
| N Fluorescein | N-2_8 | gaacaaaggaggaggttgcc | C3-Fluorescein |
| N Fluorescein | N-2_9 | atcaacccaaacctccgagg | C3-Fluorescein |
| N Fluorescein | N-2_10 | tctgctgcagttctcccggg | C3-Fluorescein |
| N Fluorescein | N-2_11 | tattggatgctcactctctg | C3-Fluorescein |
| N Fluorescein | N-2_12 | ccagaagactatctaagggc | C3-Fluorescein |
| N Fluorescein | N-2_13 | tggcatggtggacccttctc | C3-Fluorescein |
| N Fluorescein | N-2_14 | atatgatgcaccccagcttt | C3-Fluorescein |
| N Fluorescein | N-2_15 | ctatcccctgcatacccaag | C3-Fluorescein |
| N Fluorescein | N-2_16 | cactgggaccaccatggatg | C3-Fluorescein |
| N Fluorescein | N-2_17 | tcttgagtgagtggcttcct | C3-Fluorescein |
| N Fluorescein | N-2_18 | ctggacttgccaggctttgg | C3-Fluorescein |
| N Fluorescein | N-2_19 | acgagttgctgctgccctgg | C3-Fluorescein |
| N Fluorescein | N-2_20 | cccgggatgagttgactcta | C3-Fluorescein |
| N Fluorescein | N-2_21 | aagctaaaggaggggaatcc | C3-Fluorescein |
| N Fluorescein | N-2_22 | tggaggctctcatcaacaag | C3-Fluorescein |
| N Fluorescein | N-2_23 | gtcgaaagaaggcaaagcaa | C3-Fluorescein |
| N Fluorescein | N-2_24 | cccggaggatgatgatgaaa | C3-Fluorescein |
| N Fluorescein | N-2_25 | gctctgactcgtggcaacaa | C3-Fluorescein |
| N Fluorescein | N-2_26 | tgccaagaaaatgattgttc | C3-Fluorescein |
| N Fluorescein | N-2_27 | gtggggctgactgggagaag | C3-Fluorescein |
| N Fluorescein | N-2_28 | atcgaactcttaaagcagta | C3-Fluorescein |
| N Fluorescein | N-2_29 | aaggatttgatgcccgtagg | C3-Fluorescein |
| N Fluorescein | N-2_30 | tgggtccgagagtttgctta | C3-Fluorescein |
| N Fluorescein | N-2_31 | gtggaccgcaatgagattga | C3-Fluorescein |
| N Fluorescein | N-2_32 | gatccagtttgctgctcaag | C3-Fluorescein |
| N Fluorescein | N-2_33 | tggacaactatcaagagctt | C3-Fluorescein |
|  |  |  |  |
| **Probe set** | **Sequence Name** | **Sequence** | **Label** |
| Gc Q670 | Gc_1 | ccaccaagaaagcctcatag | Quasar 670 |
| Gc Q670 | Gc_2 | caggcctctctaaaatgtgg | Quasar 670 |
| Gc Q670 | Gc_3 | gctctttttccttcttatat | Quasar 670 |
| Gc Q670 | Gc_4 | tttgcctgtatgtagcatta | Quasar 670 |
| Gc Q670 | Gc_5 | ggcctcttaagactatactc | Quasar 670 |
| Gc Q670 | Gc_6 | tctttgactggttttctgga | Quasar 670 |
| Gc Q670 | Gc_7 | gggggaatcaacagttgtga | Quasar 670 |
| Gc Q670 | Gc_8 | ccatttgatgataggcgaga | Quasar 670 |
| Gc Q670 | Gc_9 | aagggaaccctgatagctat | Quasar 670 |
| Gc Q670 | Gc_10 | tcttgtgatggagatgagcg | Quasar 670 |
| Gc Q670 | Gc_11 | ggtagaggaggagtttatgt | Quasar 670 |
| Gc Q670 | Gc_12 | tactacacttcactgtacct | Quasar 670 |
| Gc Q670 | Gc_13 | ctgcatatagttcttccatc | Quasar 670 |
| Gc Q670 | Gc_14 | ctctctgcccacaataaaga | Quasar 670 |
| Gc Q670 | Gc_15 | cacatccacaggaactggaa | Quasar 670 |
| Gc Q670 | Gc_16 | ccgccttcttaaatttgaca | Quasar 670 |
| Gc Q670 | Gc_17 | ttttgaggtggactttgtgg | Quasar 670 |
| Gc Q670 | Gc_18 | ctctgtacaggctgatctaa | Quasar 670 |
| Gc Q670 | Gc_19 | ggtgttcaagctttctctaa | Quasar 670 |
| Gc Q670 | Gc_20 | acctttgcagcttcaaaagg | Quasar 670 |
| Gc Q670 | Gc_21 | ctgccacagacaaggaatga | Quasar 670 |
| Gc Q670 | Gc_22 | tttgttgtctttgagagggg | Quasar 670 |
| Gc Q670 | Gc_23 | tagatcagttggagtgcaca | Quasar 670 |
| Gc Q670 | Gc_24 | tcttatttcatacaagccca | Quasar 670 |
| Gc Q670 | Gc_25 | tcatgaatcatgccttaggg | Quasar 670 |
| Gc Q670 | Gc_26 | ggagatcaggtgcaattcag | Quasar 670 |
| Gc Q670 | Gc_27 | tcagaaattcctcggcaagg | Quasar 670 |
| Gc Q670 | Gc_28 | atgcaattgttgatgagcca | Quasar 670 |
| Gc Q670 | Gc_29 | tcaggctcaaacagcttttc | Quasar 670 |
| Gc Q670 | Gc_30 | tcacaaactggggttcagtt | Quasar 670 |
| Gc Q670 | Gc_31 | aatagacctgggagcatcat | Quasar 670 |
| Gc Q670 | Gc_32 | tgactttgatggctctgttt | Quasar 670 |
| Gc Q670 | Gc_33 | tgggtgcataaactcactct | Quasar 670 |
| Gc Q670 | Gc_34 | aaagaggcccttagagtttt | Quasar 670 |
| Gc Q670 | Gc_35 | gcttatttgtgcacacgtat | Quasar 670 |
| Gc Q670 | Gc_36 | gtgtttcaatgtgaacccat | Quasar 670 |
| Gc Q670 | Gc_37 | tttgagcagtgtggaggatg | Quasar 670 |
| Gc Q670 | Gc_38 | tttgttggggaaagcacgac | Quasar 670 |
| Gc Q670 | Gc_39 | gaataggtgtctgtcttgga | Quasar 670 |
| Gc Q670 | Gc_40 | atcttgtcggggaatgtcat | Quasar 670 |
| Gc Q670 | Gc_41 | ctttagccctaaatgtctga | Quasar 670 |
| Gc Q670 | Gc_42 | ctgtctcaagtgagctatcg | Quasar 670 |
| Gc Q670 | Gc_43 | aaagggggtcaaggaagacc | Quasar 670 |
| Gc Q670 | Gc_44 | tgtctggcacagcattaatc | Quasar 670 |
| Gc Q670 | Gc_45 | tccacagaaggtgtcaacac | Quasar 670 |
| Gc Q670 | Gc_46 | gaactgattcaggcaagctc | Quasar 670 |
| Gc Q670 | Gc_47 | ctcatatgcatcagcatgtt | Quasar 670 |
| Gc Q670 | Gc_48 | agcacatacctaatgctact | Quasar 670 |
